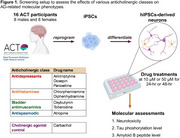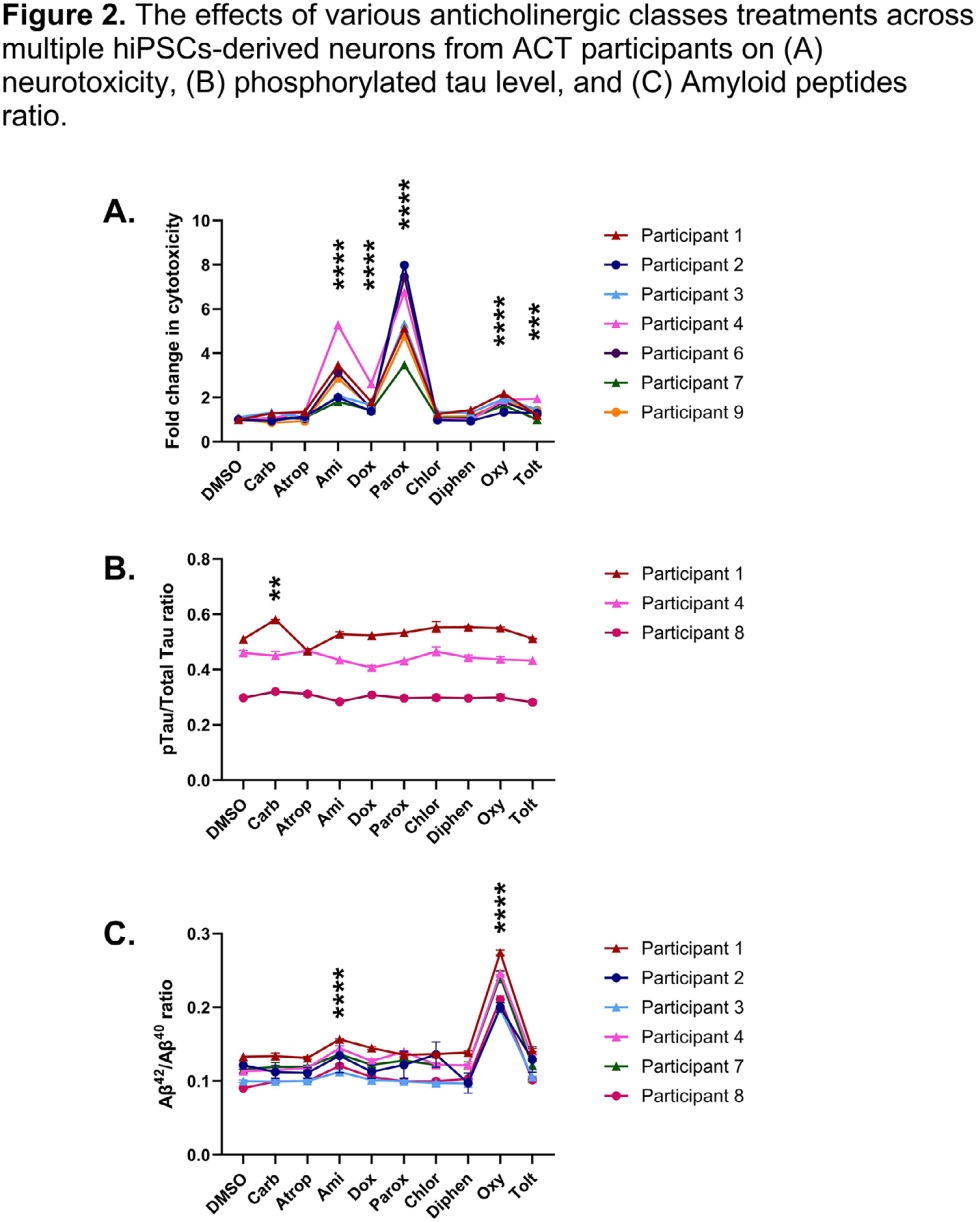# Evaluating neurotoxicity and AD‐related molecular phenotypes to anticholinergic medications in hiPSC‐derived neurons from the ACT cohort

**DOI:** 10.1002/alz70855_099906

**Published:** 2025-12-23

**Authors:** Inez K.A. Pranoto, Katherine W. Hui, Tiara A. Schwarze‐Taufiq, Paul K Crane, Shelly L Gray, Jessica E. Young

**Affiliations:** ^1^ Institute for Stem Cell and Regenerative Medicine, Seattle, WA, USA; ^2^ University of Washington, Seattle, WA, USA; ^3^ Kaiser Permanente Washington Health Research Institute, Seattle, WA, USA; ^4^ University of Washington School of Pharmacy, Seattle, WA, USA

## Abstract

**Background:**

Anticholinergic medications remain commonly prescribed to older adults, despite mounting evidence linking them to increased dementia risk. Pharmacoepidemiology studies from various populations, including the Adult Changes in Thought Study (ACT) cohort, report associations between specific anticholinergic classes – antidepressants and bladder antimuscarinics – and increased dementia incidence. However, it is difficult to determine whether these associations are directly caused by the neurotoxic effects of anticholinergic drugs or by the underlying health conditions which the medications are taken for, known as confounding by indication. To address this, we leverage human induced pluripotent stem cells‐derived‐neurons, generated from the ACT participants, to directly examine the effects of various anticholinergic classes on dementia‐related cellular phenotypes and the molecular mechanisms of how these drugs modulate neuronal functions.

**Method:**

We generated 16 hiPSC lines from ACT participants, differentiated them to cortical neurons (hiPSC‐Ns), and treated them with drugs from anticholinergic classes linked to dementia (antidepressants and bladder antimuscarinics) and those that are not associated with dementia (antihistamines and antispasmodics). All treatments were performed at two concentrations and two timepoints. To evaluate the effects of anticholinergics, we assessed molecular hallmarks of Alzheimer's disease (AD) by measuring the levels of neurotoxicity, secreted amyloid‐beta peptide ratios, and tau phosphorylation.

**Result:**

We observed that only drugs in the antidepressant and bladder antimuscarinic groups induced neurotoxicity in a time‐ and concentration‐dependent manner. Additionally, treatment with antidepressants and bladder antimuscarinics led to an increased Aβ^42^/Aβ^40^ ratio, suggesting more pathogenic processing of amyloid precursor protein (APP). Oxybutynin, a bladder antimuscarinic drug, induced the highest Aβ^42^/Aβ^40^ ratio, predominantly by increasing pathogenic Aβ^42^ peptide levels. However, no detectable changes in the phosphorylated tau‐to‐total tau ratio were observed following treatment with any of the anticholinergic drugs tested. These findings are consistent across all hiPSC‐N lines, regardless of genetic background or gender.

**Conclusion:**

Our findings reveal that antidepressants and bladder antimuscarinic drugs consistently induce neurotoxicity and pathogenic amyloid‐beta peptide secretion without affecting tau phosphorylation. Our study validates the direct correlation between increased dementia risk and the action of antidepressant and bladder antimuscarinic drugs and provides molecular insights into how these drugs induce neuronal dysfunction contributing to AD development.